# Anaesthetic management of a child with “cor–triatriatum” and multiple ventricular septal defects – A rare congenital anomaly

**DOI:** 10.4103/0019-5049.65375

**Published:** 2010

**Authors:** Sriram Sabade, Anand Vagrali, Sharan Patil, Praveen Kalligudd, Vithal Dhulked, M D Dixit, Mohan Gan, A Dayal

**Affiliations:** 1 Consultant Cardiac Anaesthesiologist, J. N. Medical College, Nehru Nagar, Belgaum – 590 010, Karnataka, India; 2 Department of Anaesthesiology, J. N. Medical College, Nehru Nagar, Belgaum – 590 010, Karnataka, India; 3 Director, KLES Heart Foundation, J. N. Medical College, Nehru Nagar, Belgaum – 590 010, Karnataka, India; 4 Professor, Department of CVTS, J. N. Medical College, Nehru Nagar, Belgaum – 590 010, Karnataka, India

**Keywords:** Atrial septal defect, cardiopulmonary bypass, congenital heart disease, cor-triatriatum, pulmonary arterial hypertension, ventricular septal defect

## Abstract

Cor-triatriatum is a rare congenital cardiac anomaly. It accounts for 0.1% of congenital heart diseases. Its association with multiple ventricular septal defects (VSD) is even rarer. A five-month-old baby was admitted with respiratory distress and failure to thrive. Clinical examination revealed diastolic murmur over mitral area. Chest X-ray showed cardiomegaly. Haematological and biochemical investigations were within normal limits. Electrocardiogram showed left atrial enlargement. 2D echo showed double-chambered left atrium (cor-triatriatum), atrial septal defect (ASD) and muscular VSD with moderate pulmonary arterial hypertension. The child was treated with 100% oxygen, diuretics and digoxin and was stabilized medically. We used balanced anaesthetic technique using oxygen, air, isoflurane, fentanyl, midazolam and vecuronium. Patient was operated under cardiopulmonary bypass (CPB) with moderate hypothermia. Through right atriotomy abnormal membrane in the left atrium was excised to make one chamber. VSD were closed with Dacron patches and ASD was closed with autologous pericardial patch. Patient tolerated the whole procedure well and was ventilated electively for 12h in the intensive care unit. He was discharged on the 10^th^ postoperative day.

## INTRODUCTION

Cor–triatriatum is a rare congenital cardiac anomaly and accounts for 0.1% of all the patients with congenital heart disease.[1] It is characterized by the presence of a fibromuscular membrane in the left atrium creating a proximal chamber which receives all the pulmonary veins and a distal chamber communicating with the mitral valve.[2] The abnormal membrane has one or several small openings communicating with the mitral valve. This results in supravalvular stenosis. The size of the orifice in the abnormal membrane determines the severity of the condition.[3] The incidence of multiple ventricular septal defects (VSD) is less than 1% in all patients with congenital heart diseases.[4] Its association with Cor–triatriatum is still rare.[5]

## CASE REPORT

**Figure 1:**
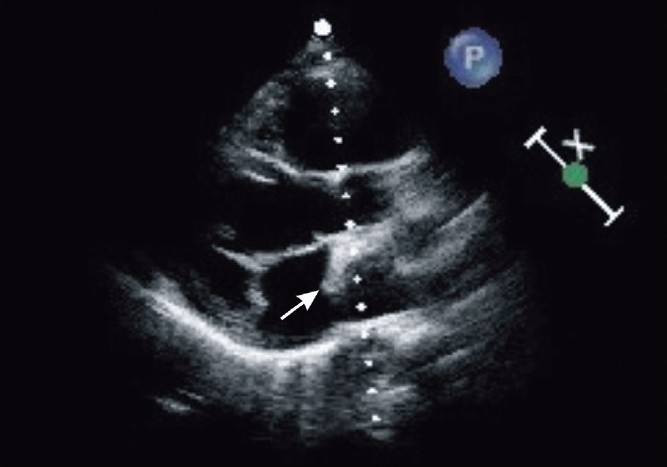
Transthoracic 2D echo: parasternal long axis view showing Cor–triatriatum

A five-month-old male child weighing 5.2 kg was admitted with respiratory distress and failure to thrive. On clinical examination, pulse was 120/min and regular, respiratory rate 40/min and blood pressure 90/60 mm Hg. On auscultation a diastolic murmur was heard at the mitral area. The chest X-ray showed cardiomegaly with increased bronchovascular markings in both the lung fields. The electrocardiogram (ECG) showed left atrial enlargement with right ventricular hypertrophy. Transthoracic 2D echo demonstrated large ASD with left to right shunting, double-chambered left atrium and mid-muscular VSD [Figure 1]. The child was treated with 100% oxygen, diuretics digoxin and was stabilized medically. Cardiac catheterization was performed to know the size and site of VSD, pulmonary artery pressure and to rule out any other associated lesions. It revealed multiple VSD, cor–triatriatum, ASD and moderate pulmonary arterial hypertension (47/9 mmHg with a mean of 25 mmHg). Child was scheduled for excision of abnormal membrane in left atrium, ASD and VSD closure under balanced general anaesthesia.

On the day of surgery, the child received intramuscular ketamine (7.5 mg), midazolam (0.25 mg) and glycopyrrolate (25 mcg) as premedication and 100% oxygen by face mask. Inside the operation theatre the child was placed on a warming mattress on the operating table. Peripheral venous access was obtained using 22-gauge cannula. Induction of anaesthesia was achieved with fentanyl (10 mcg) and additional titrated doses of midazolam. Nasotracheal intubation was facilitated by vecuronium (0.75 mg). Anaesthesia was maintained with oxygen, air, isoflurane, fentanyl, midazolam and vecuronium. A 22-G 10-cm long single-lumen catheter was inserted in the left femoral artery for continuous blood pressure monitoring, blood gas and serum electrolytes analysis. A 4.5-F triple-lumen catheter was advanced into the right internal jugular vein for monitoring central venous pressure, infusions of inotropes and vasodilators. Injection methyl prednisolone (150 mg) was given slowly after induction. Pulmonary artery (PA) pressures were recorded before going on cardiopulmonary bypass (CPB) and were 50% of systemic pressure i.e. 38/24 (mean 28) mmHg.

After median sternotomy 20 mg of heparin was administered for anticoagulation. Ascending aorta and bi-caval cannulation was done. CPB was instituted with a maximum flow of 0.9 L/min. Patient was cooled to 28°C (nasopharyngeal temperature). Blood cardioplegia was used four times. The total CPB time was 130 min and aortic cross-clamp time was 103 min. Right atriotomy revealed multiple VSD (three in number, one subaortic and two muscular), a moderate-sized ostium secundum ASD and an abnormal membrane in the left atrium. After complete excision of membrane, ASD was closed with autogenous pericardial patch. The VSD were repaired with Dacron patches using interrupted 5/0 prolene suture. Haemofiltration was carried out during CPB to remove excessive fluid and to raise the haematocrit. Leukocyte depleter (PAL) was made use of during blood transfusion intraoperatively.

Adequacy of perfusion during CPB was assessed by arterial pressure, urine output, arterial blood gases and mixed venous oxygen tension. A 22-G single-lumen 10-cm catheter was inserted into the PA through the right ventricle to monitor PA pressure throughout the intra- and postoperative period. After the completion of repair the patient was rewarmed to 37°C. He was weaned from CPB. Dobutamine, milrinone, phenoxybenzamine and nitroglycerin were used during the weaning process. Hyperkalaemia was corrected. Blood glucose was monitored every hour. After weaning from CPB residual heparin was neutralized with 30 mg of protamine sulfate. The PA pressures were between 35-40% of systemic pressure in the postoperative period. The patient was electively ventilated for 12 h using pressure-regulated volume control mode of ventilation in the intensive therapy unit. He was discharged from the hospital on the 10^th^ postoperative day.

## DISCUSSION

Cor-triatriatum is an unusual congenital anomaly with a reported incidence of 0.1–4.0% in all patients with congenital heart diseases.[6] In classic cor-triatriatum a membranous partition having the shape of a wind sock separates the more proximal chamber which receives the pulmonary veins from the more distal left atrium that communicates with the mitral valve. The wind sock is directed towards the mitral valve. The communicating orifice in the membrane ranges from less than 3 mm to about 1 cm in diameter.[7] The anomalous septum contains cardiac muscle fibres which are occasionally calcified. The more distal true left atrium communicates with the atrial appendage. Occasionally, a patent foramen ovale or ASD allows the lower left atrial chamber to communicate with the right atrium.[8]

In other variations of cor-triatriatum the accessory chamber communicates with both the left atrium and the right atrium. It may communicate with the systemic circulation, i.e. superior vena cava via an anomalous venous connection; the accessory chamber may communicate only with the right atrium or it may communicate with the systemic circulation i.e. portal vein via an anomalous vein.

Other associated anomalies may be ASD, VSD, coarctation of aorta (COA), Tetralogy of Fallot (TOF), asplenia, polysplenia. Classic cor–triatriatum where there is no alternative pathway for pulmonary venous blood, the stenotic opening in the membrane results in supravalvular mitral stenosis with all the features of elevated pulmonary venous pressure which are transmitted to the lungs, causing pulmonary oedema, the features that determine the condition of the patient. If the opening is 3 mm in diameter or less, the symptoms occur in infancy and are similar to those of total anomalous pulmonary venous return with obstruction. If the opening is larger, the symptoms manifest later in infancy, childhood or occasionally later in life.[9]

Most patients with this anomaly succumb within the first few months of life, though exceptionally patients with an adequate size of orifice in the membrane may survive until their teens and even into adulthood. Death occurs with pulmonary oedema, pneumonia or right-sided failure.

The only appropriate treatment is surgery. Medical treatment is indicated initially for those patients who are in heart failure. Surgery should have every chance of success with excellent prognosis. Pulmonary arterial changes are reversible. In this case cor–triatriatum was associated with ASD and multiple VSD and features of pulmonary vascular obstruction.[10] Oelert *et al*., reported successful surgical treatment of cor–triatriatum in a four-and-a-half-month-old infant, similar in age to the present case.[11] Ingrid and Hollinger *et al*.,[12] have opined that cor–triatriatum should be managed like mitral stenosis.

The anaesthetic goal for such cases is to prevent exacerbation of pulmonary artery hypertension (PAH) and hemodynamic instability. PAH is managed by adequate ventilatory support, inodilators (milrinone and dobutamine) and pulmonary dilators (nitroglycerin, phenoxybenzamine). Pain and anxiety are known to cause catecholamine release which in turn can adversely influence pulmonary vascular tone. Ketamine has been used in critical case of mitral stenosis. It has been reported that ketamine does not adversely affect pulmonary artery pressure.[13] We used intramuscular ketamine for its sedative and analgesic effects. Thiopentone, propofol, inhalational agents are myocardial depressants. Fentanyl, midazolam and muscle relaxant-based anaesthesia has proved to be suitable in PAH. Inhaled nitric oxide and prostaglandin are beneficial in reducing the PAH.[14] PAH crisis is the main concern in the postoperative period and is treated with 100% oxygen with moderate hyperventilation; treatment of both respiratory and metabolic acidosis, removal or attenuation of precipitating factors should be undertaken. Inodilators, vasodilators and sidanfil citrate are also advocated.

To conclude, we report here the anaesthetic management of a male child who presented with cor-triatriatum, ASD and multiple VSD which is a rare finding. Understanding the pathophysiology and haemodynamics of combined or complex cardiac disease of this nature is important in the management of anaesthesia. Fentanyl and midazolam-based anaesthetic technique and appropriate management of PAH helped us in the perioperative management of this patient.
